# Novel diagnostic markers for periprosthetic joint infection: a systematic review

**DOI:** 10.3389/fcimb.2023.1210345

**Published:** 2023-07-17

**Authors:** Melanie Schindler, Nike Walter, Guenther Maderbacher, Irene K. Sigmund, Volker Alt, Markus Rupp

**Affiliations:** ^1^ Department of Trauma Surgery, University Hospital Regensburg, Regensburg, Germany; ^2^ Department of Orthopaedic Surgery, University Hospital of Regensburg, Asklepios Klinikum Bad Abbach, Bad Abbach, Germany; ^3^ Nuffield Orthopaedic Centre, Oxford University Hospitals National Health Service (NHS) Foundation Trust, Oxford, United Kingdom

**Keywords:** periprosthetic joint infection, PJI, diagnostic marker, diagnostics, synovial fluid

## Abstract

**Background:**

Identifying novel biomarkers that are both specific and sensitive to periprosthetic joint infection (PJI) has the potential to improve diagnostic accuracy and ultimately enhance patient outcomes. Therefore, the aim of this systematic review is to identify and evaluate the effectiveness of novel biomarkers for the diagnosis of PJI.

**Methods:**

We searched the MEDLINE, EMBASE, PubMed, and Cochrane Library databases from January 1, 2018, to September 30, 2022, using the search terms “periprosthetic joint infection,” “prosthetic joint infection,” or “periprosthetic infection” as the diagnosis of interest and the target index, combined with the term “marker.” We excluded articles that mentioned established biomarkers such as CRP, ESR, Interleukin 6, Alpha defensin, PCT (procalcitonin), and LC (leucocyte cell count). We used the MSIS, ICM, or EBJS criteria for PJI as the reference standard during quality assessment.

**Results:**

We collected 19 studies that analyzed fourteen different novel biomarkers. Proteins were the most commonly analyzed biomarkers (nine studies), followed by molecules (three studies), exosomes (two studies), DNA (two studies), interleukins (one study), and lysosomes (one study). Calprotectin was a frequently analyzed and promising marker. In the scenario where the threshold was set at ≥50-mg/mL, the calprotectin point-of-care (POC) performance showed a high sensitivity of 98.1% and a specificity of 95.7%.

**Conclusion:**

None of the analyzed biomarkers demonstrated outstanding performance compared to the established parameters used for standardized treatment based on established PJI definitions. Further studies are needed to determine the benefit and usefulness of implementing new biomarkers in diagnostic PJI settings.

## 1 Introduction

Periprosthetic joint infection (PJI) is a devastating complication that can occur after total joint arthroplasty (TJA). It often requires long-term antibiotic therapy and multiple revision surgeries, and even with optimal treatment, it can significantly decrease a patient’s quality of life. Additionally, the risk of mortality is high for those affected by PJI (Wildeman et al., 2021). In addition to the significant impact on individual patients, the socioeconomic burden of PJI is substantial and expected to increase in the future (Peel et al., 2013) The one-year and five-year risk of PJI after total hip arthroplasty (THA) is 0.7% and 1.1%, respectively, while for total knee arthroplasty (TKA), the corresponding figures are 0.7% and 1.4% (Kurtz et al., 2018). The overall one-year and five-year survival rates after PJI diagnosis were 88.7% and 67.2% for THA and 91.7% and 71.7% for TKA, respectively (Kurtz et al., 2018). In Germany, the total number of total joint arthroplasty (TJA) procedures is projected to increase by 45% for TKA and 23% for THA between now and 2040 (Rupp et al., 2020). Similarly, in the US, the incidence rate of primary TKAs is projected to increase by 43% from 2020 to 2050 (Klug et al., 2021). Among these revision surgeries, the biggest share is performed due to PJI (25%), followed by mechanical loosening (19%) as the second most common reason for TKA revisions (Bozic et al., 2015).

The treatment of infected and aseptic failures after TJA differs significantly and can impose a significant burden on patients (Andersson et al., 2010). Therefore, it is essential to avoid treating a non-infected joint as an infected one, and vice versa, as this can lead to increased morbidity, unnecessary costs, and avoidable surgical interventions (Moojen et al., 2014). Accurate diagnostics are thus of paramount importance in clinical practice to ensure appropriate treatment and avoid these adverse outcomes (Kurtz et al., 2022). In some cases, diagnosing PJI is straightforward, as clear clinical findings such as the presence of a sinus tract or pus around the implanted prosthesis are considered confirming diagnostic criteria (Parvizi et al., 2018; McNally et al., 2021). However, in many cases, these confirming criteria are not present, making PJI diagnostics challenging (Balato et al., 2020). Diagnosis typically relies on laboratory tests such as serology or synovial fluid analysis, microbiological analysis of tissue specimens or synovial fluid, as well as histological and radiographic findings. In recent years, efforts have been made to improve diagnostic accuracy. In 2011, the Musculoskeletal Infection Society (MSIS) published PJI criteria that classify “major” criteria, including the presence of a communicating sinus tract and two positive periprosthetic cultures, and “minor” criteria, such as elevated ESR/CRP, elevated synovial leukocyte count, elevated synovial polymorphonuclear (PMN)%, purulent material, isolated organism in one culture, and intraoperative frozen sections with histology (Parvizi et al., 2011). In 2013, the Infectious Diseases Society of America (IDSA) provided its own PJI diagnostic criteria with the aim of standardizing diagnostics (Osmon et al., 2013). Unlike the MSIS criteria, the IDSA criteria do not include elevated inflammatory markers but consider other factors such as the growth of a virulent organism from a single culture or the presence of acute inflammation from histopathology of the periprosthetic tissue. In 2013, the International Consensus Meeting (ICM) introduced a new minor criterion - the leukocyte esterase in synovial fluid measured by a urine strip test. Later, Parvizi et al. updated the ICM concept by introducing a scoring system based on the different sensitivity and specificity of the markers in 2018 (Parvizi et al., 2018). This updated system included promising new markers such as alpha-defensin in synovial fluid and D-dimer in serum. In 2021, the European Bone and Joint Infection Society (EBJIS) criteria were introduced, classifying cases as “unlikely,” “likely,” and “confirmed” All these criteria rely on various clinical, laboratory, microbiological, and histological analyses, as well as intraoperative findings, to establish a diagnosis (see **Table 1**).

**Table 1:** Commonly used PJI criteria and clinical and diagnostic markers included.

	MSIS (Parvizi et al., 2011) (2011)	IDSA (Osmon et al., 2013) (2013)	ICM (Parvizi and Gehrke, 2014) (2013)	ICM (Parvizi et al., 2018) (2018)	EBJIS (McNally et al., 2021) (2021)
Clinical					
Communicating sinus tract	✓	✓	✓	✓	✓
Purulent material	✓	✓	X	✓	✓
Blood					
CRP (mg/L)	↑	X	10	10	>10
ESR (mm/hr)	↑	X	30	30	X
D-Dimer (µg/L)	X	X	X	30	X
Synovial fluid cytological analysis					
Synovial leukoycte count (cells/µL)	✓	X	3.000	3.000	>1.500
Synovial PMN (%)	↑	X	90	70	>65%
Synovial fluid biomarkers					
Alpha Defensin	X	X	X	1.0	✓
Leukocyte esterase	X	X	+/++*	++*	X
Microbiology					
Culture	≥ 1	≥ 1	✓	✓	≥ 1
Sonication (CFU/ml)	X	X	X	X	>1
Histology					
High-power field (400 x magnificantion)	>5 neutrophils per hpf in 5 phf	✓	>5 neutrophils per hpf in 5 phf	✓	>5 neutrophils in single hpf
Others					
Nuclear imaging (WBC scintigraphy)	X	X	X	X	**✓**

(CRP- C-reactive protein, ESR- erythrocyte sedimentation rate, PMN- polymorphonuclear neutrophils, WBC- white blood cell count).

* The leukocyte concentration is evaluated using test strips on the basis of the color scale from left - to right +++.

Despite the improvements made in recent decades that have made correct diagnosis of PJI more likely through the introduction of different PJI criteria, the identification of a novel biomarker that is highly specific and sensitive for PJI could enable easier and more accurate diagnosis of this devastating disease, ultimately improving patient outcomes. Therefore, the objective of this systematic review is to identify and evaluate novel biomarkers for preoperative PJI diagnostics.

## 2 Materials and methods

### 2.1 Search strategy

This systematic review of the literature was performed according to the preferred Reporting Items for Systematic Reviews and Meta-Analyses guidelines (Page et al., 2021).We searched in the electronic databases MEDLINE, EMBASE, PubMed and Cochrane Library. The following search terms were used to screen literature that utilized new marker for PJI diagnosis: We used “periprosthetic joint infection” OR “prosthetic joint infection” OR “periprosthetic infection” as the diagnosis of interest and the target index applied AND “marker”. To focus on novel biomarkers already used biomarkers of the established PJI diagnostic criteria of MSIS, ICM and EBJIS were not included in the analysis. Therefore, the search terms included NOT “CRP”, NOT “ESR”, NOT “Interleukin 6”, NOT “Alpha defensin”, NOT “PCT” (Procalcitonin), NOT “LC” (leucocyte cell count). A second approach to only include novel biomarkers was setting the time frame for study inclusion from January 1, 2018 to September 30, 2022. After identification, all records were screened by two independent reviewers for the diagnostic markers either determined from blood samples or synovial fluid. All included articles had to be published in English. Animal studies, and studies investigating histological diagnostics were excluded from the analysis.

### 2.2 Data extraction and quality assessment

Two reviewers performed data extraction independently, and divergences was discussed with a third reviewer. Data were extracted from the eligible studies including the author names, year of publication, country, total number of participants (PJI/control group), mean age of the patients, level of evidence, study design, biomarker, sample type, sample part, sample collection, reference standard and sensitivity, specificity or cut-off of the new marker.

The Quality Assessment of Diagnostic Accuracy Studies-2 (QUADAS-2) tool was used to determine the potential risk of bias of each study following the full-text assessment (Whiting et al., 2011). MSIS, ICM or EBJS criteria for PJI were considered the reference standard during quality assessment.

An application to register this review in the International Prospective Register of Systematic Reviews (PROSPERO) was submitted but rejected because of study prioritization focusing on SARS- CoV-2 infections.

## 3 Results

### 3.1 Search results

**Figure 1:**
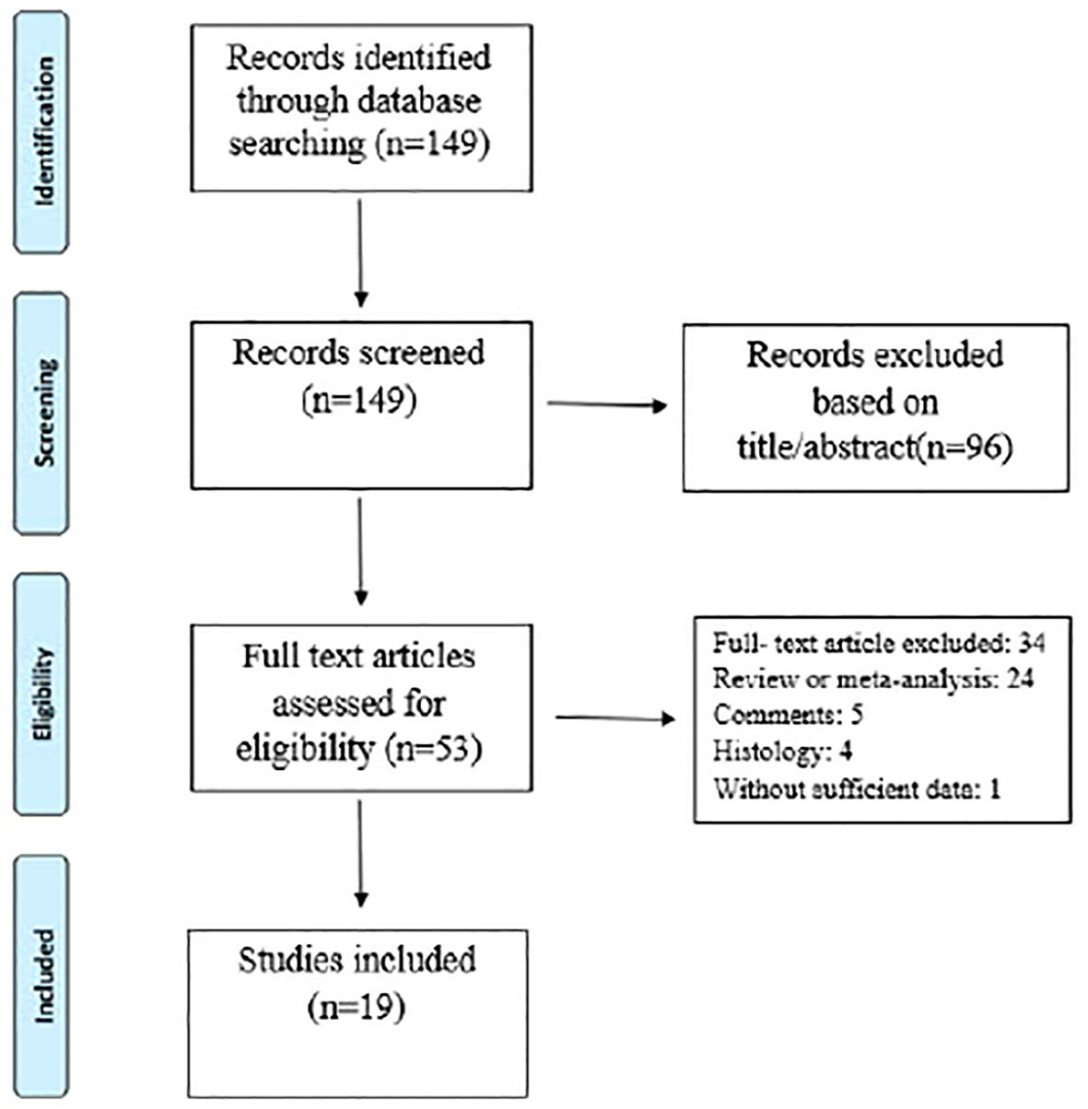
Flow diagram of study selection.

The electronic database and bibliography search identified 149 studies, of which 130 were excluded after title/abstract and full text evaluation (see **Figure 1** and **Table 2**). Therefore, 19 studies met the inclusion criteria. Of these, fifteen studies (79%) had prospective designs, and the remaining four (22%) were retrospective studies. Six studies (33%) focused solely on periprosthetic knee infections, while thirteen (67%) included both periprosthetic knee and hip infections. All studies provided diagnostic data for periprosthetic hip and knee infections based on the MSIS, EBJIS, or ICM criteria. The number of patients in the selected studies ranged from 23 to 224. Among the selected studies, 15 (79%) analyzed synovial fluid, three (16%) analyzed blood, and one (5%) analyzed urine (**Table 3**). The different biomarker analyses are shown in **Tables 4** and **5**.

**Table 2:** Summary of *subgroups* in the *diagnosis* marker.

Marker	Number of studies
Protein	9
Calprotectin	Grassi et al. (2022), Warren et al. (2022), Warren et al. (2021) (3)
LTF, MNDA, PRTN3	Wang et al. (2019) (1)
NGAL	Dijkman et al. (2020) (1)
Urinary peptide markers	Omar et al. (2021) (1)
Lipocalin-2	Vergara et al. (2019) (1)
Soluble Pecam-1	Fuchs et al. (2021) (1)
Zonulin, LPS, sCD14	Chisari et al. (2022) (1)
sCD28, sCD80, sCTLA-4, sBTLA	Jubel et al. (2021) (1)
Exosomes	2
EVs	Rüwald et al. (2020), Sallai et al. (2022) (2)
Interleukins	1
Cytokines (IL-1b, IL-2, IL-4, IL-5, IL-6, IL-8, IL-10, IL-12, IL-17, GM-CSF, TNF-α, INF-γ)	Fröschen et al. (2020) (1)
Lysosomes	1
MPO	Ikeda et al. (2020) (1)
DNA	2
Cf-DNA	Echeverria et al. (2021), Cobra et al. (2022) (2)
Molecule	3
BJI InoPlexT	Dartus et al. (2021) (1)
D-lactate	Karbysheva et al. (2020), Yermak et al. (2019) (2)

**Table 3:** Characteristics of the studies involved in the current study.

Study	Country	Patient Number (PJI)	Control group	Mean Age (y)	Level of evidence	Study design	Biomarker	Sample type	Sample Part	Sample collection	Ref Standard
Chisari et al. (Chisari et al., 2022)	USA	134 (44)	AIF 90 TJA 26	68	II	Prospective	Zonulin sCD14	Blood	Hip Knee	Before antibiotics	ICM criteria
Cobra et al. (Cobra et al., 2022)	Bra	66 (40- chronic)	AIF 26	71/70	I	Prospective	cf-DNA	Synovial fluid	Knee	Intraoperative	ICM criteria
Dartus et al. (Dartus et al., 2021)	Fra	24 (8-chronic)	AIF 16	63	IV	Retrospective	BJI InoPlexT	Blood	Hip Knee	Preoperative	MSIS criteria
Dijkman et al. (Dijkman et al., 2020)	Nld	76 (13)	AIF 89	69	III	Retrospective	NGAL LE WBC	Synovial fluid	Knee	Pre-/intraoperative	MSIS/ Pro implantat criteria
Echeverria et al. (Echeverria et al., 2021)	USA	53 (53)	0	68	II	Prospective	cf DNA	Blood	Hip Knee	Preoperative	MSIS criteria
Fröschen et al. (Fröschen et al., 2020)	Ger	32 (14)	AIF 18	68	IV	Retrospective	Cytokine	Synovial fluid	Hip Knee	Pre-/intraoperativ	MSIS criteria
Fuchs et al. (Fuchs et al., 2021)	Ger	58 (22)	AIF 20 TJA 16	71	II	Prospective	Soluble Pecam-1	Synovial fluid	Knee	Intraoperative	EBJIS criteria
Grassi et al. (Grassi et al., 2022)	Ita	93 (39)	AIF 50	77	II	Prospective	Calprotectin rapid test, Calprotectin ELISA immunoassay	Synovial fluid	Knee	Preoperative	ICM criteria
Ikeda et al. (Ikeda et al., 2020)	Jpn	37 (19- chronic)	AIF 18	75	II	Prospective	MPO	Synovial fluid	Hip, Knee	Intraoperative	ICM criteria
Jubel et al. (Jubel et al., 2021)	Ger	99 (39)	AIF 24 TJA 23 Native 13	67	II	Prospective	Soluble immunregulartory markers	Synovial fluid	Hip, Knee	Preoperative/intraoperative	MSIS criteria
Karbysheya et al. (Karbysheva et al., 2020)	Ger	224 (71)	AIF 153	66	I	Prospective	D-lactate	Synovial fluid	Hip, Knee	Preoperative	MSIS criteria/Institutional Criteria
Omar et al. (Omar et al., 2021)	Ger	30 (20)	AIF 10	70	I	Prospective	Urinary peptide markers	Urine	Hip, Knee	Preoperative	MSIS criteria
Rüwald et al. (Rüwald et al., 2020)	Ger	23 (12)	AIF 11	71	II	Prospective	EVs	Synovial fluid	Hip, Knee	Intraoperativ	MSIS criteria
Sallai et al. (Sallai et al., 2022)	Hun	34 (17)	AIF 17	72	II	Prospective	EVs	Synovial fluid	Hip, Knee	Intraoperativ	MSIS criteria
Vergara et al. (Vergara et al., 2019)	Esp	72 (22)	AIF 22 TJA 28	74	II	Prospective	Lipocalin-2	Synovial fluid	Hip Knee	Intraoperative	MSIS criteria
Wang et al. (Wang et al., 2019)	Chn	50 (25)	AIF 25	64/69	III	Retrospective	LTF, PRTN3, MNDA	Synovial fluid	Hip Knee	Intraoperative	MSIS criteria
Warren et al. (Warren et al., 2021)	USA	123 (53)	AIF 70	66	I	Prospective	Calprotectin POC test	Synovial fluid	Knee	Intraoperative	MSIS criteria
Warren et al. (Warren et al., 2022)	USA	123 (53)	AIF 70	66	I	Prospective	Calprotectin POC test	Synovial fluid	Knee	Intraoperative	EBJIS/MSIS/ICM criteria
Yermak et al. (Yermak et al., 2019)	Ger	148 (44)	AIF 104	70	I	Prospective	D-lactate	Synovial fluid	Hip, Knee, Shoulder	Preoperative/intraoperative	EBJIS criteria

NGAL, Neutrophil Gelatinase-Associated Lipocalin; LE, Leukocyte Esterase; WBC, White Blood Count; cf, DNA, Call-Free Deoxyribonucleic; MPO, Myeloperoxidase; EVs, Extracellular Vesicles; LTF, Lactoferrin; PRTN3, Polymorphonuclear leukocyte serine protease 3; MNDA, Myeloid Nuclear Differentiation Antigen.

**Table 4:** Analysis of biomarker for PJI diagnosis.

Study	Biomarker Cut-off	Sensitivity (95% CI)	Specificity (95% CI)	AUC (95% CI)	Accuracy (95%CI)	PLN (95% CI)	NLR (95% CI)	PPV	NPV
Cobra et al. (Cobra et al., 2022)	Cf-DNA 15 ng/mL	96.2 (80.4-99.9)	100 (91.2-100)	1.0 (0.9-1.0)					
Dartus et al. (Dartus et al., 2021)	BJI InoPlexT Positive	50	56	–		36	69		
Dijkman et al. (Dijkman et al., 2020)	LE ++	39	88						
MSIS									
MSIS	WBC count 2575 cells/μL	92	84						
MSIS	NGAL 0.7355 μg/mL	92	83						
Pro-Implant	LE ++	39	92						
Pro-Implant	WBC count 1865 cells/μL	100	97						
Pro-Implant	NGAL 0.7355 μg/mL	95	95						
Fröschen.et al. (Fröschen et al., 2020)	IL Ib >29.08 pg/mL	92.9 (66.1–99.8)	83.3 (58.6–96.4)	0.9 (0.9–1.0)		–	–		
	IL 2 >9.065	92.9 (68.5–99.6)	61.1 (38.6–79.7)	0.8 (0.7–1.0)					
	IL 4 >1.890	92.9 (66.1–99.8)	72.2 (46.5–90.3)	0.9 (0.8–1.0)					
	IL 5 >4.720	71.4 (41.9–91.6)	77.8 (52.4–93.6)	0.8 (0.6–1.0)					
	IL 6 >1975	92.9 (66.1–99.8)	88.9 (65.3–98.6)	1.0 (0.9–1.0)					
	IL 8 >2748	85.7 (57.2–98.2)	72.2 (46.5–90.3)	0.9 (0.7–1.0)					
	IL10 >10.38	92.9 (66.1–99.8)	83.3 (58.6–96.4)	0.9 (0.8–1.0)					
	IL12 >14.10	100.0 (76.8–100.0)	66.7 (41.0–86.7)	0.8 (0.6–0.9)					
	IL17 >124.6	92.9 (66.1–99.8)	83.3 (58.6–96.4)	0.9 (0.8–1.0)					
	GM-CSF >1.895	78.6 (49.2–95.3)	66.7 (41.0–86.7)	0.7 (0.6–0.9)					
	TNF-α >29.39	71.4 (41.9–91.6)	77.8 (52.3–93.6)	0.8 (0.6–1.0)					
	IFN-γ >6.215	92.9 (66.1-99.8)	61.1 (35.8–82.7)	0.8 (0.7–1.0)					
Grassi et al. (Grassi et al., 2022)	Calprotectin ELISA immunoassay	92.3 (79.1-98.4)	100 (92.8-100)	1.0 (0.9-1.0)		–	0.1 (0.0-0.2)	100	94.3 (84.9-98)
	Calprotectin rapid test	97.4 (86.5-99.9)	94 (83.5-98.7)	1.0 (0.9-1.0)		16.2 (5.4-48.7)	0.0 (0.0-0.2)	92.7 (80.9-97.4)	97.9 (87.1-99.7)
	LE test	46.1 (30.1-62.8)	94 (83.5-98.7)	0.7 (0.6-0.8)		7.7 (2.4-24.3)	0.6 (0.4-0.8)	85.7 (65.6-95)	73 (62.6-81.9)
Ikeda et al. (Ikeda et al., 2020)	MPO 40.000 ng/mL	84	100	–		1	0.9		
	30.000 ng/mL	95	100	–		1	0.9		
	20.000 ng/mL	95	94	–		0.9	0.9		
	10.000 ng/mL	100	94	–		1	1		
	1000 ng/mL	100	72	–		0.8	1		
	Ideal 1487- 16,463 ng/mL	100	94	1.0 (1.0–1)		95	10		
Karbysheva et al. (Karbysheva et al., 2020)	D-lactate 1.3 mmol/L MSIS	94.3 (86.2-98.4)	78.4 (66.8-81.2)	0.9 (0.9-1.0)				67 (56.9-76.1)	96.8 (91.9-99.1)
	Institutional Criteria	92.4 (84.9-96.9)	88.6 (81.9-93.5)	1.0 (0.9-1.0)				85 (76.5-91.3)	94.4 (88.7-97.7)
Omar et al. (Omar et al., 2021)	Urinary peptide markers	95	90	1.0 (0.8-1.0)				65	
Vergara et al. (Vergara et al., 2019)	Lipocalin-2 152 ng/mL	100 (88-100)	100 (94-100)	1.0 (1.0- 1.00)					
Wang et al. (Wang et al., 2019)	LTF 221.19 ng/mL	97.1	90	1		–	–		
	MNDA 13.12 ng/mL	77.1	97.5	1		–	–		
	PRTN3 7.30 ng/mL	88.6	45	1		–	–		
Warren et al. (Warren et al., 2022)	Calprotectin POC test ≥50 mg/L	98.1	95.7	1		94.5	98.5		
	≥14-mg/L	98.1	87.1	0.9		85.2	98.4		
Warren et al. (Warren et al., 2021)	Calprotectin POC test MSIS >50 mg/L	98.1	95.7	1		94.5	98.5		
	ICM	98.2	98.5	0.984		98.2	98.5		
	EBJIS	93.2	100	0.966		100	94.2		
Yermak et al. (Yermak et al., 2019)	D-lactate 1.263 mmol/l	86.4 (75-95)	80.8 (73-88)	0.93 (86-95)					

**Table 5:** Analysis of biomarker for PJI diagnosis.

Study	Subject	Biomarker	PJI	Non PJI	Sig.
Chisari et al. (Chisari et al., 2022)	PJI 44 AIF 90	Zonulin (ng/mL)	7.6± 6.1	4.6± 3.8	p < 0.001
		sCD14 (ng/mL)	555.7± 216.7	396.9± 247.9	p < 0.003
	Acute (n=14) vs. Chronic (n=30)	Zonulin (ng/mL)	11.6± 6.7	5.8± 4.8	p < 0.005
Echeverria et al. (Echeverria et al., 2021)	Pathogen identified by blood cfDNA-seq (n=35)	Species identified by surgical joint culture	23		–
		Genus identified by surgical joint culture	8		–
		Pathogen not identified by surgical joint culture	4		–
	Pathogen not identified by blood cfDNA-seq (n=15)	Species identified by surgical joint culture	12		–
		Genus identified by surgical joint culture	3		–
Fuchs et al. (Fuchs et al., 2021)	PJI vs. AIF	Soluble Pecam-1 (ng/mL)	73.0± 22.9	44.0 ± 11.8	p < 0.001
	PJI- TJA		73.0± 22.9	26.02± 6.48	p < 0.001
Jubel et al., (Jubel et al., 2021)	Soluble immunregulartory markers –	sLAG-3 (pg/ml)	319.7± 38.4	6534.3± 753.3	p < 0.001
	PJI vs. CO	sCTLA-4 (pg/ml)	450.0± 58.5	59.3± 16.9	p < 0.001
		sCD27 (pg/ml)	32088.4± 5436.8	5610.2± 2444.6	p < 0.001
		sCD80 (pg/ml)	1671.9± 184.8	238.2± 66.2	p < 0.001
		sTIM-3 (pg/ml)	319.7± 38.4	6534.3± 753.3	p < 0.001
		sPD-1 (pg/ml)	253.7± 59.4	32.8± 15.3	p < 0.001
		IDO (pg/ml)	1892.8± 519.1	38.5± 16.1	p < 0.001
		sBTLA (pg/ml)	3716.6± 674.9	594.9± 199.1	p < 0.001
Rüwald et al. (Rüwald et al., 2020)	EVs	Nanovesicles Size (nm)	224.8 ± 90.7	156.5 ± 64.4	p = 0.001
			Higher particle concentrations in PJI than AIF		p = 0.032
			High fluorescence intensities of CD 9 in AIF than PJI		p < 0.001
			High fluorescence intensities of CD 81 in AIF than PJI		p = 0.037
Sallai et al. (Sallai et al., 2022)	Polymorphonuclear derived EVs	Concentration	Higher in PJI than AIF		p = 0.0105
		Annexin V	Increased Eventnumber in PJI than AIF		p = 0.046
		Annexin V and anti-CD177	Increased Eventnumber in PJI than AIF		p = 0.0105
		Lactotransferrin	Significant difference in the abundance in PJA than AIF		p = 0.00646
		Myeloperoxidase	Significant difference in the abundance in PJA than AIF		p = 0.01061
		Lysozyme C	Significant difference in the abundance in PJA than AIF		p = 0.04687
		Annexin A6	Significant difference in the abundance in PJA than AIF		p = 0.03921
		Alpha-2-HS-glycoprotein	Significant difference in the abundance in PJA than AIF		p = 0.03146

The quality of all selected studies was evaluated using the QUADAS-2 tool, and the results are presented in **Table 6**.

**Table 6:** Quality evaluation of selected studies.

Study	Risk of bias				Applicability concerns		
	Patient selection	Index test	Reference standard	Flow and timing	Patient selection	Index test	Reference standard
Chisari et al. (Chisari et al., 2022)	High	Low	Low	Low	High	Low	Low
Cobra et al. (Cobra et al., 2022)	Low	Low	Low	Low	Low	Low	Low
Dartus et al.(Dartus et al., 2021)	High	High	Low	Low	High	High	Low
Dijkman et al. (Dijkman et al., 2020)	High	High	Low	Low	High	Low	Low
Echeverria et al., (Echeverria et al., 2021)	Low	Low	Low	Low	Low	Low	Low
Fröschen.et al. (Fröschen et al., 2020)	High	Low	Low	Low	High	Low	High
Fuchs et al.(Fuchs et al., 2021)	High	Low	Low	Low	High	Low	Low
Grassi et al. (Grassi et al., 2022)	High	High	Low	Low	High	Low	Low
Ikeda et al.(Ikeda et al., 2020)	High	High	Low	Low	High	High	Low
Jubel et al. (Jubel et al., 2021)	High	Low	Low	Low	High	Low	Low
Karbysheva et al. (Karbysheva et al., 2020)	Low	High	Low	Low	Low	Low	Low
Omar et al. (Omar et al., 2021)	Low	Low	Low	Low	Low	Low	Low
Rüwald et al.(Rüwald et al., 2020)	High	Low	Low	Low	High	Low	Low
Sallai et al. (Sallai et al., 2022)	High	Low	Low	Low	High	Low	Low
Vergara et al. (Vergara et al., 2019)	High	High	Low	Low	High	Low	Low
Wang et al. (Wang et al., 2019)	Low	Low	Low	Low	Low	Low	Low
Warren et al. (Warren et al., 2022)	Low	Low	Low	Low	Low	Low	Low
Warren et al. (Warren et al., 2021)	Low	Low	Low	Low	Low	Low	Low
Yermak et al. (Yermak et al., 2019)	High	Low	Low	Low	High	Low	Low

**Figure 2:**
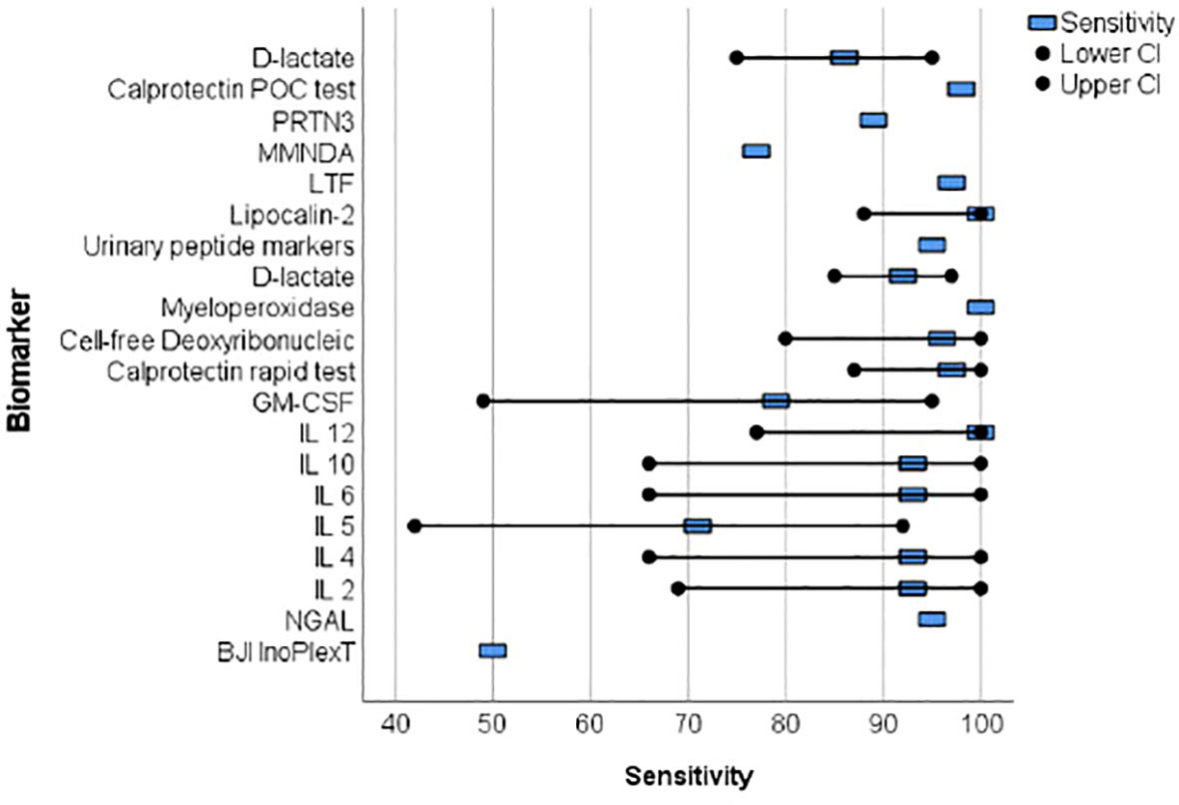
The forest plots of the sensitivity for novel diagnosis marker of periprosthetic joint infection.

**Figure 3:**
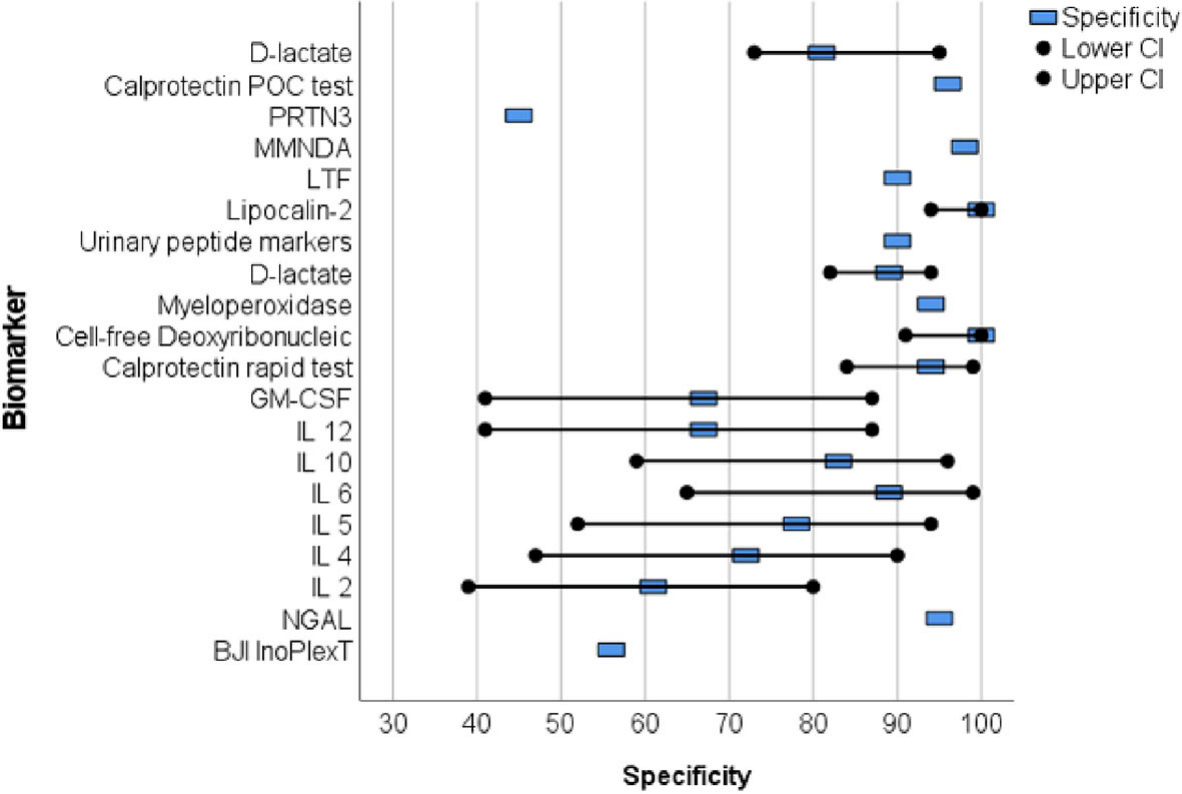
The forest plots of the specificity for novel diagnostic marker of periprosthetic joint infection.

Proteins were primarily analyzed as potential markers, with calprotectin being a frequently studied novel marker (Warren et al., 2021; Grassi et al., 2022; Warren et al., 2022) (**Table 2**). In one study, the calprotectin point-of-care (POC) performance showed a sensitivity of 98.1% and a specificity of 95.7% in a scenario with a threshold of ‡50-mg/mL (**Figures 2**, **3**) (Warren et al., 2021).

Wang et al. collected 50 synovial fluid aspirates from hips and knees and verified the most promising proteins using ELISA (enzyme-linked immunosorbent assay) (Wang et al., 2019). The study identified that lactoferrin (LTF), myeloid nuclear differentiation antigen (MNDA), and polymorphonuclear leukocyte serine protease 3 (PRTN3) were sensitive, while LTF and MNDA were specific for diagnosing PJI. A retrospective cohort study analyzed only TKA synovial fluid and when applying the MSIS criteria, neutrophil gelatinase-associated lipocalin (NGAL) revealed 92% sensitivity and 83% specificity (Dijkman et al., 2020) (**Figures 2**, **3**).

An additional novel approach to diagnose PJI involves analyzing the pattern of urinary peptide excretion. In a study analyzing urinary samples from 30 patients prior to surgery, a marker model consisting of 83 peptides demonstrated the best diagnostic performance with a sensitivity of 95% and specificity of 90% for diagnosing PJI (Omar et al., 2021) (**Figures 2**, **3**). In a study by Vergara et al., synovial fluid was collected from 30.6% of patients with proven infections, 30.6% with aseptic implant failures, and 38.8% controls. Lipocalin-2 (LCN2) was found to discriminate nearly perfectly between controls and confirmed infections (Vergara et al., 2019) (**Figure 2**, **3**). Soluble Pecam 1 (sPecam-1) is an immunologically reactive molecule that is removed from the surface of T cells upon activation by proinflammatory signals. Synovial samples were taken intraoperatively from 16 native knees, 20 aseptic knee revisions, and 22 knees with PJI. The amount of sPecam-1 was significantly greater in knees with PJI compared to aseptic TKA revision procedures (p ≤ 0.001) (Fuchs et al., 2021) (**Table 5**). In a prospective cohort study, Zonulin, soluble CD14 (sCD14), and lipopolysaccharide (LPS) were tested in blood samples before antibiotic administration. The study included 134 patients, of which 44 had PJI, 64 had aseptic failure, and 26 underwent primary TKA. Zonulin (7.642 ± 6.077 ng/mL vs 4.560 ± 3.833 ng/mL; p < 0.001) and sCD14 levels (555.721 ± 216.659 ng/mL vs 396.872 ± 247.920 ng/mL; p = 0.003) were significantly increased in PJI compared to non-infected cases (Chisari et al., 2022) (**Table 5**). Jubel et al. analyzed fourteen soluble immunoregulatory markers using bead-based multiplex assays and showed significant differences for nine markers when comparing PJI and control groups (Jubel et al., 2021) (**Table 5**). Extracellular vesicles (EVs) represent another group of the novel markers analyzed (Rüwald et al., 2020; Sallai et al., 2022). The concentration of EVs was significantly higher in the septic samples (p = 0.0105) and showed a different size pattern as compared to the aseptic ones (**Table 4**). Fröschen et al. evaluated a combination of six cytokines (IL-2, IL-4, IL-5, IL-6, lL-12 and GM-CSF) performed better in diagnosing chronic PJI than any cytokine alone. Regression analysis for this combination revealed a sensitivity of 100% and a specificity of 88.9% for a cut-of value of 0.41 (Fröschen et al., 2020). Myeloperoxidase (MPO) is a bactericidal enzyme that acts against pathogenic microorganisms, such as in PJI. In a small cohort study of 37 patients, MPO levels were significantly higher in the chronic PJI group than in the aseptic failure group (p < 0.001) (p < 0.001) (Ikeda et al., 2020) (**Table 5**). Another marker is cell-free deoxyribonucleic acid (cf-DNA) in synovial fluid and peripheral blood (Echeverria et al., 2021; Cobra et al., 2022). The sensitivity and specificity in synovial fluid were 96.2% and 100%, respectively (**Figures 2**, **3**). BJI InoPlex is a multiplex ELISA that measures the immune response to certain bacterial species from *Staphylococcus epidermidis, aureus* and *lugdunensis, Streptococcus B* and *Cutibacterium acnes*. Dartus et al. included eleven hip and thirteen knee arthroplasty cases. The sensitivity for diagnosing a chronic PJI based on the 2018 ICM criteria was 50% and the specificity was 56% (Dartus et al., 2021) (**Figure 2**, **3**).

D-lactate was studied in the largest cohort of patients (148 and 224) by Karbysheva et al. and Yermak et al. who used different PJI criteria. The sensitivity ranged from 86.4% to 94.3% and the specificity ranged from 78.4% to 80.8% with similar cutoffs (Yermak et al., 2019; Karbysheva et al., 2020) (**Figures 2**, **3**).

## 4 Discussion

Over the last five years, 19 studies have reported on new biomarkers for PJI, with 15 of these studies specifically focused on parameters in the synovial fluid. Most of the studies analyzed proteins (nine studies), followed by molecules (three studies), exosomes (two studies), DNA (two studies), interleukins (one study), and lysosomes (one study). Calprotectin is a promising and frequently analyzed marker (**Table 1**) (Warren et al., 2021; Grassi et al., 2022; Warren et al., 2022). In scenarios with a threshold of ≥50 mg/mL, the calprotectin point-of-care performance showed a high sensitivity of 98.1% and specificity of 95.7% (**Figures 2**, **3**). LCN2 is another hopeful marker that nearly perfectly discriminates between controls and confirmed infections in a small cohort of patients (72 patients/22 PJI) (**Figures 2**, **3**) (Vergara et al., 2019). D-lactate, which has been analyzed in a large cohort of patients, is also noteworthy, revealing 94% sensitivity and 78% specificity (**Figures 2**, **3**) (Yermak et al., 2019; Karbysheva et al., 2020). The sensitivity and specificity of these markers are comparable to those of established markers. A review by Sigmund et al. presented the performance of established and novel serum inflammatory biomarkers. The sensitivity and specificity of established markers such as erythrocyte sedimentation rate (ESR) or white blood cell count (WBC) demonstrated similar sensitivity and specificity in comparison to new markers. C-reactive protein (CRP) with a cut-off of 3-32mg/L showed a sensitivity of 62-100% and specificity of 64-96%, while procalcitonin demonstrated a maximum sensitivity of 90% and specificity of 100% (Sigmund et al., 2021).

More than two-thirds of the studies analyzed biomarkers from synovial fluid, but it’s important to note that diagnostic hip aspirations are unsuccessful in up to one-third of patients (Christensen et al., 2022). Five of the 19 studies analyzed only preoperative aspirates. Furthermore, there is a discordance of approximately 20% between preoperative aspirate culture and intraoperative synovial fluid culture, which makes relying solely on synovial fluid in the preoperative setting for diagnosing PJI challenging (Li et al., 2021). A meta-analysis of 14 studies that pooled preoperative aspiration culture data revealed an average sensitivity of 67.6% (range 28% to 100%) and an average specificity of 98.4% (range 96% to 100%) (Rodriguez-Merchan, 2018).

Inflammation triggers a series of signaling cascades, and different markers investigated in PJI are linked to these cascades, either up or down. For instance, calprotectin is secreted by neutrophils (Stríz and Trebichavský, 2004) which play a vital role in PJI diagnostics as PMN%. Likewise, the measurement of calprotectin in synovial fluid is significantly associated with PMN% and is an important marker for diagnosing PJI (Burri et al., 2013; Lisowska-Myjak et al., 2016; Honar et al., 2022). Similarly, D-lactate concentration is linked to microbial load. The concentration of D-lactate seems to depend on the number of bacteria, as higher levels of D-lactate were observed in culture-positive PJI compared to culture-negative PJI (Yermak et al., 2019). Given the interdependence of markers in the inflammation signaling cascades, it is not surprising that relying on a single new marker alone may not revolutionize PJI diagnostics.

Alpha defensin, which is a diagnostic marker included in the ICM 2018 criteria (**Table 1**), was initially hailed as a game-changing diagnostic marker. However, as it became known that alpha defensin is released by neutrophilic granulocytes and acts as part of the non-specific immune system, it was not surprising that the hoped-for diagnostic breakthrough was followed by disappointment. In the literature, the sensitivity of alpha-defensin for the diagnosis of PJI has been reported to range from 67% to 100%, and the specificity from 89% to 99% (Kasparek et al., 2016; Wyatt et al., 2016; Balato et al., 2020). Renz et al. calculated a sensitivity of 84% using the MSIS criteria, 67% using the IDSA criteria, and 54% using the PRO-IMPLANT/EBJIS criteria (Renz et al., 2018). Such variation according to the various criteria presents a challenge in clinical application. Therefore, the routine use of alpha-defensin testing is not recommended in the literature and should only be performed as an additional diagnostic test. Costs also have to be taken into account when using alpha-defensin for diagnostics. ELISA for alpha-defensin is much more expensive than the leukocyte esterase test strip (£0.11 [US$0.17] per test), costing around £500 [US$760] per test (Wyatt et al., 2016).

Besides cost, availability is a major concern for implementing new biomarkers in clinical practice. Established markers like CRP and synovial WBC are commonly used in medical centers, whereas newer markers like alpha defensin are rarely used for routine diagnosis of PJI. Alpha defensin is only collected in 19.4% of cases, while microbiological (97.7%), leukocyte count (74.8%), and PMN% (65.8%) are the most frequently measured parameters in diagnostic setting (Ahmad et al., 2016). Furthermore, storage of specimens poses a challenge as certain markers, such as cytokines and s-Pecam1, require specific temperatures during transportation, which can complicate logistics. As a result, introducing these markers in clinical practice can be difficult.

Modern genomic sequencing diagnostics may offer a solution to the challenges associated with biomarkers and conventional microbial diagnostics. While culture-based detection methods remain the gold standard, they are plagued by several limitations, including low sensitivity. Microbiological culture only detects the pathogen in 44-80% of cases (Malhotra and Morgan, 2004; Williams et al., 2004). One major factor that significantly affects the probability of detecting a pathogen through culture-based methods is the duration of the culture (Saleh et al., 2003; Schäfer et al., 2008). Additionally, contamination and resulting false positive findings can also be problematic (Yee et al., 2013). To overcome these limitations, culture-independent, molecular biology-based methods can be employed as an alternative diagnostic tool. In particular, plasmatic detection of circulating free DNA through Next Generation Sequencing (NGS) has shown promise as a diagnostic method for patients with bloodstream infections. Metagenomic NGS (mNGS) offers the ability to identify multiple organisms in a single sample (Gu et al., 2019). Early studies have suggested that NGS-based diagnostics are more effective than conventional culture-based methods for detecting bloodstream infections (Grumaz et al., 2016; Decker et al., 2017; Grumaz et al., 2019). In the case of PJI, Echeverria et al. identified the pathogen in 35 cases, including four cases that were deemed culture-negative (57%) (Echeverria et al., 2021). Having a pathogenic marker such as circulating free DNA could be beneficial as it specifically identifies present bacteria compared to nonspecific markers. Thus, NGS could be utilized to identify the pathogen in cases where culture-based methods are ineffective.

Several limitations of this systematic review must be acknowledged. First, the study compared three different PJI criteria, which are the most commonly used ones. The MSIS and ICM criteria were used in six studies. Sigmund et al. conducted a retrospective study of 206 PJI patients, of which 101 (49%) were diagnosed with PJI using the EBJIS definition, 99 (48%) with the IDSA definition, and 86 (42%) with the ICM definition. A total of 84 cases (41%) had an infection based on all three criteria. The novel EBJIS definition appears to be more sensitive for PJI diagnosis compared to the IDSA and ICM definitions. All infections classified by the IDSA or ICM criteria were identified by the EBJIS definition, indicating that the EBJIS definition is superior to the IDSA and ICM criteria for PJI diagnostics. However, only two studies in this systematic review used the EBJIS definition, which was introduced recently in 2021 (Sigmund et al., 2022). However, the present systematic review is limited by several factors. Secondly, only two studies in our review employed the recently introduced EBJIS definition, which limits the generalizability of our findings. Thirdly, the use of different cut-off values for biomarkers across studies makes comparison challenging. Finally, the limited availability of information on the time elapsed since the arthroplasty procedure may affect the accuracy of some biomarkers, as their diagnostic performance may vary in the early postoperative period (Yi et al., 2014).

## 5 Conclusion

Based on the current analysis, no novel biomarker investigated in the past five years for diagnosing PJI has been proven to outperform the already established diagnostic parameters. Further studies may demonstrate the usefulness of additional markers, such as calprotectin, in the established PJI diagnostic criteria.

## Author contributions

SM, NW, and MR contributed to conception and design of the study. SM and NW organized the database. SM and NW performed the statistical analysis. SM wrote the first draft of the manuscript. NW and MR wrote sections of the manuscript. All authors contributed to the article and approved the submitted version.
